# Assessing risk factors of acute kidney injury and its influence on adverse outcomes after lung transplantation: methodology is important

**DOI:** 10.1080/0886022X.2021.1936039

**Published:** 2021-06-07

**Authors:** Bin Hu, Fu-Shan Xue, Shao-Hua Liu, Yi Cheng

**Affiliations:** Department of Anesthesiology, Beijing Friendship Hospital, Capital Medical University, Beijing, People's Republic of China

Dear Editor,

By a retrospective analysis, Du et al. [1] assessed the incidence and risk factors of early-onset acute kidney injury (AKI) during the first week after lung transplantation and its association with postoperative mortality. They showed that a higher baseline estimated glomerular filtration rate (eGFR) was associated with the occurrence of early postoperative AKI. Just like the authors had described in the discussion, this is a counterintuitive finding. It is generally believed that a decreased baseline eGFR is a well-established predisposing factor of postoperative AKI and even mild preoperative kidney dysfunction is significant with an increased risk of postoperative AKI [2]. The authors raised that this counterintuitive finding might be attributable to glomerular hyperfiltration, i.e., a state of little kidney reserve capacity which is intolerable to nephrotoxic insults and prone to the occurrence of AKI. Other than the limitations described by the authors in the discussion, however, we noted several methodological issues that would have confounded their findings.

First, the study objects are patients with end-stage lung disease undergoing lung transplantation. The authors provided the preoperative comorbidities of patients, but not an assessment of overall health status and acute illness conditions before surgery. The available evidence indicates that preoperative higher acute physiologic assessment and chronic health evaluation (APACHE) II scores are the independent risk factors of AKI in patients undergoing lung transplantation [3]. Furthermore, a higher lung allocation score at transplantation, preoperative mean pulmonary artery pressures greater than 35 mm Hg, ICU stay before transplantation and preoperative use of extracorporeal membrane oxygenation or mechanical ventilation have been significantly associated with the occurrence of AKI following lung transplantation [4]. It is generally believed that patients with AKI are often sicker and have a worse respiratory outcome with a need for a bundle of treatments in the ICU before transplantation. Most important, the readers were not provided with the details of anesthetic and intraoperative management. Thus, it is difficult to determine the extent of influence that anesthesiologists’ interventions may have on the occurrence of postoperative AKI. Other than transplant types, operation duration, intraoperative extracorporeal membrane oxygenation support and blood loss provided by the authors, the recent works have shown that intraoperative increased fluid balance, use of vasoactive drugs, hypoxemia, severe arterial hypotension with hemodynamic decompensation are significantly associated with an increased risk of AKI after lung transplantation [3,5]. Indeed, multivariate analysis is useful for the identification of risk factors of adverse perioperative events by adjusting patients’ baseline characteristics and controlling selection biases in a retrospective study. To obtain the true inferences of multivariate analysis for an adjusted odds ratio of measured outcome, however, all of the known factors affecting measured outcome must be taken into the model. If a critical factor is missed, multivariate adjustment for an odds ratio of measured outcome may be biased and even a spurious association between intervention and outcome of interest is obtained [6]. As the abovementioned several important perioperative factors associated with the development of AKI after lung transplantation were not taken into the multivariate model, we argue that their results of multivariate analysis to determine the risk of AKI would be distorted.

Second, in statistical analysis, the authors described that the potential risk factors with a *p*<.20 in univariate analysis were incorporated into multivariate logistic regression in a forward linear regression stepwise fashion. According to the provided in table 3 of Du et al.’s article, it was unclear why only serum creatinine, estimated glomerular filtration rate, hemoglobin, glucose, and median tacrolimus concentration were incorporated into the univariate mode as the potential risk factors for adjustment. Generally speaking, only the variables with statistical significance in the bivariate analyses are entered into the univariate model to examine multicollinearity among candidate covariate variables [6]. Based on the results of bivariate analyses for perioperative data in tables 1 and 2 of Du et al.’s article, preoperative serum creatinine, eGFR, hemoglobin and glucose were not significantly different between patients with and without AKI. That is, according to the above principle of multivariate regression modeling, these factors should not be included in the univariate model. As calibration of the multivariate model was not performed, moreover, the readers cannot determine whether the multivariate model established in this study has a good fit [7 ].

Finally, the Cox proportional hazard model was used to determine the effects of severe AKI (stages 2 and 3) on 30-day or 1-year mortality by adjusting for potential confounders including baseline demographics, intraoperative and postoperative risk factors for AKI. According to their supplemental material, we noted that the above-mentioned important perioperative factors associated with the development of AKI after lung transplantation were not included in the model. This limitation might similarly have biased the hazard ratios of severe AKI for postoperative mortality.

## Author contributions

All authors had carefully read manuscript of Wu et al., analyzed their methods and data. BH suggested comment points and drafted this manuscript. FSX critically revised comment points and this manuscript, and is the author responsible for this manuscript. LSH and YC revised comment points and this manuscript. All authors had seen and approved the final manuscript.
